# Supplementary material for: Drug repurposing for aging research using model organisms
Source: Aging Cell. 2017 Jun 16;16(5):1006–15. doi: 10.1111/acel.12626 (PMC5595691; doi:10.1111/acel.12626)
Supplement: Supplementary file 7 — Data S1 Zip‐Archive of all report cards. [file ACEL-16-1006-s007.zip › RC_IPA.pdf]

IPA

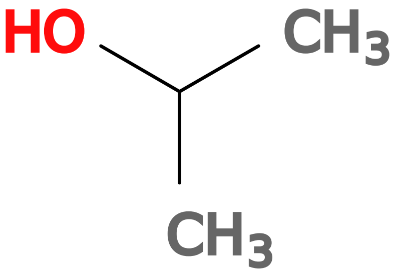

Database identifiers

ChEMBLCompound	CHEMBL582
CHEBI	17824
ZINC	ZINC00901159
eMolecules	478530

Ranking

	Rank	Score
Drosophila	476/697	0.299
C. elegans	455/591	0.107

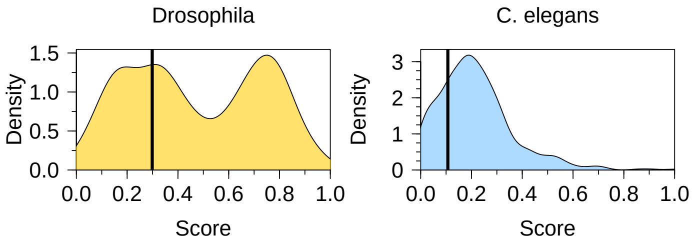

	Ageing implication	Domain conservation	Binding site conservation	Binding affinity	Bioavailability	Lipinski	Promiscuity	Purchasability	Drug approval	**Total**
Drosophila	1.0	0.971	0.995	0.282	(0.9)	0.0	-0.146	0.1	0.1	0.299
C. elegans	1.0	0.968	0.877	0.282	0.223	0.0	-0.146	0.1	0.1	0.107

Names

- **propan-2-ol**
- 1-methylethanol
- 1-methylethyl alcohol
- 2-hydroxypropane
- i-propanol
- i-Propylalkohol
- Isopropyl alcohol
- sec-propanol

Roles

protic solvent

Status

Approved drug (according to ChEMBL)	Yes
Number of Rule of 5 violations	0
Binding affinity to original target in log units (RF-Score prediction)	4.06
Burns *C. elegans* bioavailability prediction	-3.97

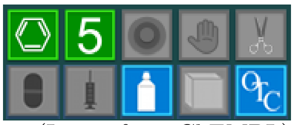

(Image from ChEMBL)

Compound Target Characteristics

Serine/threonine-protein kinase Chk1

Best gene implication in ageing for this target family came from gene O14757 annotated in UniProt release 2014_02. Annotation GO subterm of 7568 (aging) was Non-traceable Author Statement

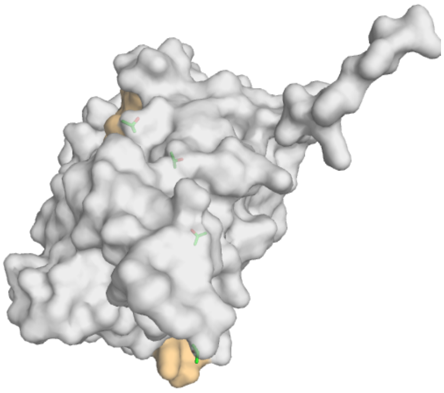

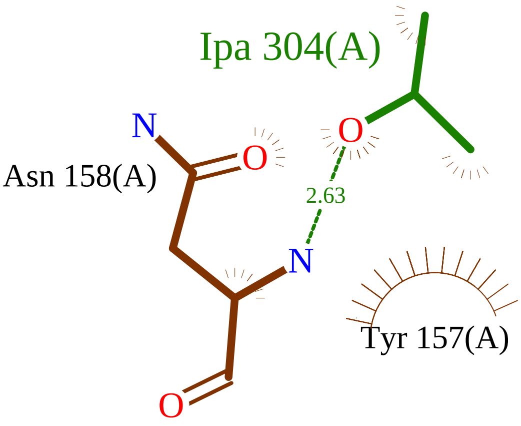

protein	amino acids contacts (binding site)
PDB:4fsm:chainA:O14757	V T E F I P Y N Y R A G E L W K H L E N S A
PDB:4fsn:chainA:O14757	V T E F I P Y N Y R A G E L W K H L E N S A
PDB:4fst:chainA:O14757	V T E F I P Y N Y R A G E L W K H L E N S A
PDB:4fsu:chainA:O14757	V T E F I P Y N Y R A G E L W K H L E N S A
PDB:4fsw:chainA:O14757	V T E F I P Y N Y R A G E L W K H L E N S A
PDB:4fsy:chainA:O14757	V T E F I P Y N Y R A G E L W K H L E N S A
PDB:4fsz:chainA:O14757	V T E F I P Y N Y R A G E L W K H L E N S A
PDB:4ft0:chainA:O14757	V T E F I P Y N Y R A G E L W K H L E N S A
PDB:4ft3:chainA:O14757	V T E F I P Y N Y R A G E L W K H L E N S A
PDB:4ft7:chainA:O14757	V T E F I P Y N Y R A G E L W K H L E N S A
PDB:4fta:chainA:O14757	V T E F I P Y N Y R A G E L W K H L E N S A
PDB:4ftc:chainA:O14757	V T E F I P Y N Y R A G E L W K H L E N S A
PDB:4fti:chainA:O14757	V T E F I P Y N Y R A G E L W K H L E N S A
PDB:4ftj:chainA:O14757	V T E F I P Y N Y R A G E L W K H L E N S A
PDB:4ftk:chainA:O14757	V T E F I P Y N Y R A G E L W K H L E N S A
PDB:4ftl:chainA:O14757	V T E F I P Y N Y R A G E L W K H L E N S A
PDB:4ftm:chainA:O14757	V T E F I P Y N Y R A G E L W K H L E N S A
PDB:4ftn:chainA:O14757	V T E F I P Y N Y R A G E L W K H L E N S A
PDB:4fto:chainA:O14757	V T E F I P Y N Y R A G E L W K H L E N S A
PDB:4ftq:chainA:O14757	V T E F I P Y N Y R A G E L W K H L E N S A
PDB:4ftr:chainA:O14757	V T E F I P Y N Y R A G E L W K H L E N S A
PDB:4ftt:chainA:O14757	V T E F I P Y N Y R A G E L W K H L E N S A
PDB:4ftu:chainA:O14757	V T E F I P Y N Y R A G E L W K H L E N S A
PDB:4gh2:chainA:O14757	V T E F I P Y N Y R A G E L W K H L E N S A
tr:J3KN87:J3KN87_HUMAN	V T E F I P Y N Y R A G E L W K H L E N S A
sp:O14757:CHK1_HUMAN	V T E F I P Y N Y R A G E L W K H L E N S A
sp:Q91ZN7:CHK1_RAT	I T E F I P H N Y R A G E L W K H L E N S A
tr:G3UYB1:G3UYB1_MOUSE	I T E F I P H N Y R A G E L W K H L E T S A
tr:G3UYC5:G3UYC5_MOUSE	I T E F I P H N Y R A G E L W K H L E T S A
sp:O35280:CHK1_MOUSE	I T E F I P H N Y R A G E L W K H L E T S A
sp:O61661:CHK1_DROME	Q T G F I P C K Y K T G E L W N R L T S G T
tr:Q965F6:Q965F6_CAEEL	K N P F L L V D Y K A A G L W K R L V N S E
tr:Q9NF25:Q9NF25_CAEEL	N N P F I P V D F K S G N L W N K L E N N E
sp:Q9N3Z3:CHK1_CAEEL	K N P F I P N K Y G A G E L W N R V D K D K
sp:P38147:CHK1_YEAST	M D P F I P R K Y S V G Q T F N R L P D N K

protein	whole protein ident	simil	domain-based ident	simil	contact-based ident	simil
PDB:4fsm:chainA:O14757	1.0	1.0	1.0	1.0	1.0	1.0
PDB:4fsn:chainA:O14757	1.0	1.0	1.0	1.0	1.0	1.0
PDB:4fst:chainA:O14757	1.0	1.0	1.0	1.0	1.0	1.0
PDB:4fsu:chainA:O14757	1.0	1.0	1.0	1.0	1.0	1.0
PDB:4fsw:chainA:O14757	1.0	1.0	1.0	1.0	1.0	1.0
PDB:4fsy:chainA:O14757	0.99	1.0	0.99	0.99	1.0	1.0
PDB:4fsz:chainA:O14757	0.99	0.99	0.99	0.99	1.0	1.0
PDB:4ft0:chainA:O14757	1.0	1.0	1.0	1.0	1.0	1.0
PDB:4ft3:chainA:O14757	0.99	0.99	0.99	0.99	1.0	1.0
PDB:4ft7:chainA:O14757	1.0	1.0	1.0	1.0	1.0	1.0
PDB:4fta:chainA:O14757	1.0	1.0	1.0	1.0	1.0	1.0
PDB:4ftc:chainA:O14757	1.0	1.0	1.0	1.0	1.0	1.0
PDB:4fti:chainA:O14757	1.0	1.0	1.0	1.0	1.0	1.0
PDB:4ftj:chainA:O14757	1.0	1.0	1.0	1.0	1.0	1.0
PDB:4ftk:chainA:O14757	1.0	1.0	1.0	1.0	1.0	1.0
PDB:4ftl:chainA:O14757	1.0	1.0	1.0	1.0	1.0	1.0
PDB:4ftm:chainA:O14757	1.0	1.0	1.0	1.0	1.0	1.0
PDB:4ftn:chainA:O14757	1.0	1.0	1.0	1.0	1.0	1.0
PDB:4fto:chainA:O14757	1.0	1.0	1.0	1.0	1.0	1.0
PDB:4ftq:chainA:O14757	1.0	1.0	1.0	1.0	1.0	1.0
PDB:4ftr:chainA:O14757	1.0	1.0	1.0	1.0	1.0	1.0
PDB:4ftt:chainA:O14757	1.0	1.0	1.0	1.0	1.0	1.0
PDB:4ftu:chainA:O14757	1.0	1.0	1.0	1.0	1.0	1.0
PDB:4gh2:chainA:O14757	0.99	0.99	0.99	0.99	1.0	1.0
tr:J3KN87:J3KN87_HUMAN	0.91	0.91	1.0	1.0	1.0	1.0
sp:O14757:CHK1_HUMAN	1.0	1.0	1.0	1.0	1.0	1.0
sp:Q91ZN7:CHK1_RAT	0.94	0.98	0.98	1.0	0.91	0.95
tr:G3UYB1:G3UYB1_MOUSE	0.84	0.89	0.97	0.99	0.86	0.93
tr:G3UYC5:G3UYC5_MOUSE	0.87	0.91	0.97	0.99	0.86	0.93
sp:O35280:CHK1_MOUSE	0.93	0.98	0.97	0.99	0.86	0.93
sp:O61661:CHK1_DROME	0.44	0.75	0.56	0.87	0.45	0.62
tr:Q965F6:Q965F6_CAEEL	0.2	0.39	0.37	0.73	0.41	0.64
tr:Q9NF25:Q9NF25_CAEEL	0.22	0.47	0.39	0.79	0.41	0.67
sp:Q9N3Z3:CHK1_CAEEL	0.31	0.63	0.48	0.8	0.41	0.57
sp:P38147:CHK1_YEAST	0.23	0.63	0.36	0.74	0.27	0.6

grp (FBgn0261278) associated phenotypes

cell cycle defective, chemical sensitive, decreased cell death, maternal effect, mitotic cell cycle defective, non-rescuable maternal effect, partially lethal - majority die, radiation resistant, radiation sensitive

(Information from FlyBase)

grp (UniProt:O61661) annotation

Function: Serine/threonine-protein kinase which is required for checkpoint-mediated cell cycle arrest and activation of DNA repair in response to the presence of DNA damage or unreplicated DNA. May also negatively regulate cell cycle progression during unperturbed cell cycles. May phosphorylate the CDC25 phosphatase stg, which promotes its degradation. This results in increased inhibitory tyrosine phosphorylation of CDC2-cyclin complexes and consequent inhibition of cell cycle progression. (PubMed:10209095, PubMed:10469601, PubMed:10980701, PubMed:12919679, PubMed:14711410, PubMed:15723794, PubMed:15860729, PubMed:16079276, PubMed:7925016, PubMed:9197245, PubMed:9214509).

Subcellular location: Nucleus ECO:0000269—PubMed:10980701, PubMed:16079276).

Developmental stage: Expressed both maternally and zygotically. Maternally supplied mRNA is degraded during progression from nuclear stage 12 to nuclear stage 13. Zygotic expression is seen at reduced levels later in embryogenesis and during larval development. Higher expression is seen in pupae, coincident with ovarian differentiation. May be activated during the syncytial blastoderm divisions which precede cellularization, the Drosophila equivalent of the midblastula transition (MBT). Developmentally regulated activation of the DNA replication checkpoint may occur as the nucleo-cytoplasmic ratio increases and maternal replication factors are depleted. Elongation of the

embryonic cell cycle may allow time for the transcription of genes that initiate the switch from maternal to zygotic control of embryogenesis. (PubMed:9197245, PubMed:9214509).
Ptm: Phosphorylated in a MEI-41/ATR dependent manner in response to DNA damage or the presence of unreplicated DNA. (PubMed:15860729).

(Information from UniProt)

chk-1 (WBGene00000498) associated phenotypes
lethal, locomotion variant, mid larval lethal, sterile

(Information from WormBase)

chk-1 (UniProt:Q9N3Z3) annotation
Function: Serine/threonine-protein kinase which is required for checkpoint-mediated cell cycle arrest and activation of DNA repair in response to the presence of DNA damage or unreplicated DNA. May also negatively regulate cell cycle progression during unperturbed cell cycles. Required for checkpoint mediated cell cycle arrest in response to DNA damage in germline cells. Essential for embryogenesis. (PubMed:15326393).
Subcellular location: Cytoplasm Nucleus
Tissue specificity: Expressed in the germline. (PubMed:15326393).
Developmental stage: Highly expressed in the embryo. (PubMed:15326393).

(Information from UniProt)

GTPase HRas

Best gene implication in ageing for this target family came from gene P01112 annotated in UniProt release 2014_02. Annotation GO subterm of 7568 (aging) was Inferred from Direct Assay

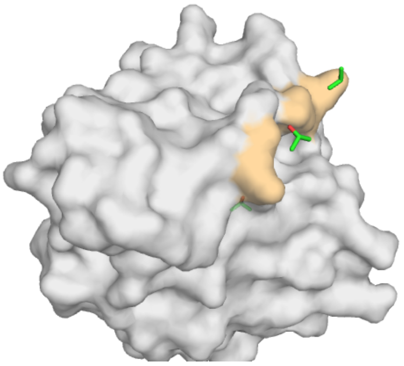

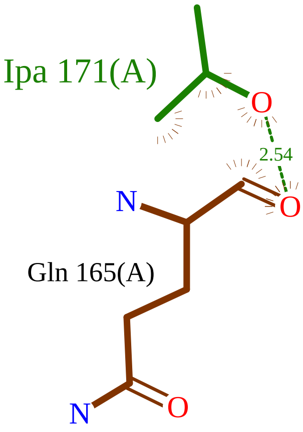

protein	amino acids contacts (binding site)			
PDB:1p2u:chainA:P01112	K	D	D	Q
sp:P01111:RASN_HUMAN	K	D	D	Q
tr:Q5U091:Q5U091_HUMAN	K	D	D	Q
sp:P01112:RASH_HUMAN	K	D	D	Q
sp:P20171:RASH_RAT	K	D	D	Q
sp:Q04970:RASN_RAT	K	D	D	Q
sp:Q61411:RASH_MOUSE	K	D	D	Q
tr:Q9D091:Q9D091_MOUSE	K	D	D	Q
tr:Q4FJP3:Q4FJP3_MOUSE	K	D	D	Q
sp:P08646:RAS1_DROME	K	E	E	K
sp:P22981:LET60_CAEEL	K	D	D	K
sp:P01119:RAS1_YEAST	N	D	Y	D
sp:P01120:RAS2_YEAST	S	D	Y	D

	whole protein		domain-based		contact-based	
protein	ident	simil	ident	simil	ident	simil
PDB:1p2u:chainA:P01112	1.0	1.0	1.0	1.0	1.0	1.0
sp:P01111:RASN_HUMAN	0.85	0.96	0.9	0.98	1.0	1.0
tr:Q5U091:Q5U091_HUMAN	0.85	0.96	0.9	0.98	1.0	1.0
sp:P01112:RASH_HUMAN	1.0	1.0	1.0	1.0	1.0	1.0
sp:P20171:RASH_RAT	1.0	1.0	1.0	1.0	1.0	1.0
sp:Q04970:RASN_RAT	0.85	0.96	0.9	0.98	1.0	1.0
sp:Q61411:RASH_MOUSE	1.0	1.0	1.0	1.0	1.0	1.0
tr:Q9D091:Q9D091_MOUSE	0.86	0.96	0.9	0.98	1.0	1.0
tr:Q4FJP3:Q4FJP3_MOUSE	0.84	0.94	0.9	0.98	1.0	1.0
sp:P08646:RAS1_DROME	0.77	0.91	0.85	0.95	0.25	0.77
sp:P22981:LET60_CAEEL	0.74	0.89	0.81	0.94	0.75	0.88
sp:P01119:RAS1_YEAST	0.34	0.52	0.61	0.87	0.25	0.41
sp:P01120:RAS2_YEAST	0.34	0.5	0.62	0.88	0.25	0.35

Ras85D (FBgn0003205) associated phenotypes

RU486 conditional, cell autonomous, cell death defective, cell non-autonomous, cell polarity defective, cell size defective, conditional, decreased cell death, decreased cell number, decreased cell size, developmental rate defective, dominant, endocytosis defective, female semi-sterile, germline clone, heat sensitive, hyperplasia, increased cell death, increased cell growth, increased cell number, increased cell size, large body, learning defective, lethal - all die before end of P-stage, lethal - all die before end of pupal stage, majority die during P-stage, maternal effect, melanotic mass phenotype, mitotic cell cycle defective, neoplasia, neuroanatomy defective, neurophysiology defective, partially, partially lethal - majority die, short lived, size defective, small body, somatic clone, some die during P-stage, some die during pharate adult stage, some die during pupal stage, temperature conditional, terminal phenotype, tumorigenic

(Information from FlyBase)

Ras85D (UniProt:P08646) annotation

Function: May mediate a signal that determines the fate of photoreceptor cells in the developing compound eye. Ras proteins bind GDP/GTP and possess intrinsic GTPase activity.
Enzyme regulation: Alternates between an inactive form bound to GDP and an active form bound to GTP. Activated by a guanine nucleotide-exchange factor (GEF) and inactivated by a GTPase- activating protein (GAP).
Subcellular location: Cell membrane (PubMed:18503409); Lipid-anchor (PubMed:18503409); Cytoplasmic side (PubMed:18503409). Note=Loss of prenylation causes protein location to the cytoplasm.
Tissue specificity: Expressed in the posterior termini of the embryo, restricted mainly to the embryonic central nervous system, and in the eye imaginal disk. (PubMed:7873789).

(Information from UniProt)

let-60 (WBGene00002335) associated phenotypes

AWA odorant chemotaxis defective, AWC odorant chemotaxis defective, L2 arrest, L3 arrest, antibody staining reduced, antibody staining variant, axon outgrowth variant, bloated, cell differentiation variant, egg laying defective, egg laying imipramine resistant, egg laying variant, germ cell development variant, gonad arm morphology variant, lethal, locomotion variant, male mating defective, mid larval lethal, multivulva, neuron degeneration, oocyte accumulation, oocytes small, organism starvation response variant, protein expression absent, reduced brood size, rod like larval lethal, sterile, unfertilized oocytes laid, vulval cell induction increased, vulvaless

(Information from WormBase)

let-60 (UniProt:P22981) annotation

Function: The level of let-60 controls the switch between vulval and hypodermal cell fates during C.elegans vulval induction. May stimulate the guanine nucleotide exchange factor (GEF) activity of rap-1.
Subunit: Interacts with soc-2. (PubMed:9674433).
Subcellular location: Cell membrane; Lipid-anchor.

(Information from UniProt)

Hepatocyte growth factor receptor

Best gene implication in ageing for this target family came from gene Q2IBC7 via mapping the annotation from RGD 3082 annotated in RGD 2014-03-11. Annotation GO subterm of 7568 (aging) was Inferred from Expression Pattern

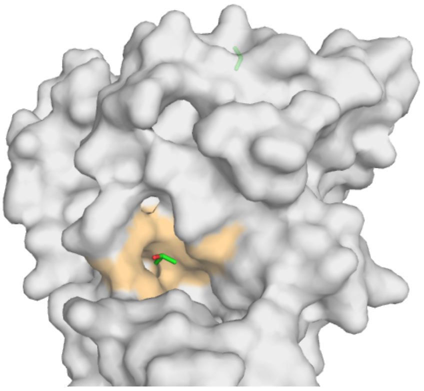

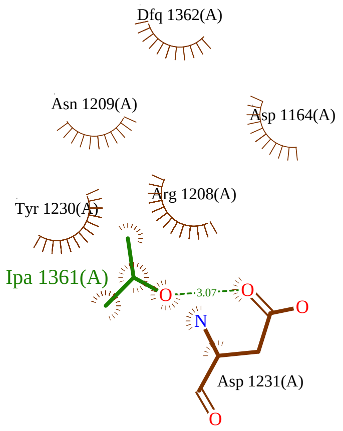

protein	amino acids contacts (binding site)
PDB:3a4p:chainA:P08581	D R N Y D Q D Y E
tr:B4DPY6:B4DPY6_HUMAN	D R N Y D Q D Y E
tr:B4DLF5:B4DLF5_HUMAN	D R N Y D Q D Y E
sp:P08581:MET_HUMAN	D R N Y D Q D Y E
tr:Q2IBC7:Q2IBC7_RAT	D R N Y D Q D Y E
tr:F8VQL0:F8VQL0_MOUSE	D R N Y D Q D Y E
tr:Q6AHP3:Q6AHP3_CAEEL	D R N - D K E Y D
tr:H1AGA1:H1AGA1_CAEEL	D R N - D K E Y D
tr:H2KZU7:H2KZU7_CAEEL	D R N - D K E Y D

protein	whole protein ident	simil	domain-based ident	simil	contact-based ident	simil
PDB:3a4p:chainA:P08581	1.0	1.0	0.99	1.0	1.0	1.0
tr:B4DPY6:B4DPY6_HUMAN	0.19	0.19	1.0	1.0	1.0	1.0
tr:B4DLF5:B4DLF5_HUMAN	0.69	0.69	1.0	1.0	1.0	1.0
sp:P08581:MET_HUMAN	1.0	1.0	1.0	1.0	1.0	1.0
tr:Q2IBC7:Q2IBC7_RAT	0.88	0.96	0.99	1.0	1.0	1.0
tr:F8VQL0:F8VQL0_MOUSE	0.89	0.96	0.99	1.0	1.0	1.0
tr:Q6AHP3:Q6AHP3_CAEEL	0.13	0.4	0.45	0.8	0.56	0.58
tr:H1AGA1:H1AGA1_CAEEL	0.13	0.4	0.45	0.8	0.56	0.58
tr:H2KZU7:H2KZU7_CAEEL	0.13	0.4	0.45	0.8	0.56	0.58

Macrophage migration inhibitory factor

Best gene implication in ageing for this target family came from gene P34884 annotated in UniProt release 2014_02. Annotation GO subterm of 7568 (aging) was Inferred from Mutant Phenotype

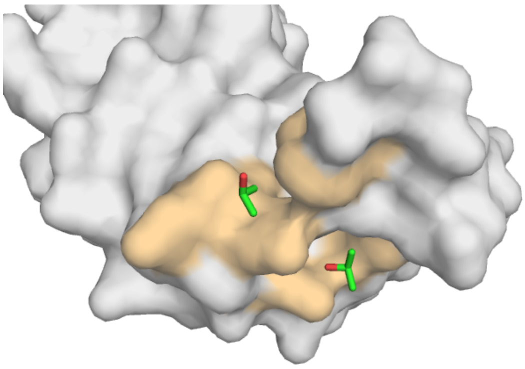

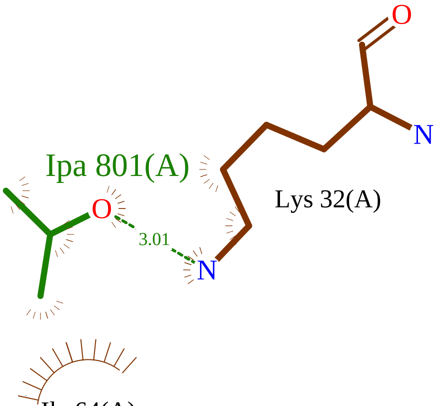

protein	amino acids contacts (binding site)
PDB:2ooz:chainA:P14174	G S E Q K Q Y I G G A Q W F
PDB:3djh:chainA:P14174	G S E Q K Q Y I G G A Q W F
PDB:3u18:chainA:P14174	G S E Q K Q Y I G G A Q W F
PDB:3wns:chainA:P14174	G S E Q K Q Y I G G A Q W F
PDB:4f2k:chainA:P14174	G S E Q K Q Y I G G A Q W F
PDB:4p01:chainA:P14174	G S E Q K Q Y I G G A Q W F
sp:P14174:MIF_HUMAN	G S E Q K Q Y I G G A Q W F
tr:I4AY87:I4AY87_HUMAN	G S E Q K Q Y I G G A Q W F
sp:P30904:MIF_RAT	G S E Q K Q Y I G G A Q W F
sp:P34884:MIF_MOUSE	G S E Q K Q Y I G G A Q W F
tr:Q545F0:Q545F0_MOUSE	G S E Q K Q Y I G G A Q W F
tr:Q9U228:Q9U228_CAEEL	E K E T K Q Y I G S A K Y F

protein	whole protein ident	simil	domain-based ident	simil	contact-based ident	simil
PDB:2ooz:chainA:P14174	1.0	1.0	1.0	1.0	1.0	1.0
PDB:3djh:chainA:P14174	1.0	1.0	1.0	1.0	1.0	1.0
PDB:3u18:chainA:P14174	1.0	1.0	1.0	1.0	1.0	1.0
PDB:3wns:chainA:P14174	1.0	1.0	1.0	1.0	1.0	1.0
PDB:4f2k:chainA:P14174	1.0	1.0	1.0	1.0	1.0	1.0
PDB:4p01:chainA:P14174	1.0	1.0	1.0	1.0	1.0	1.0
sp:P14174:MIF_HUMAN	1.0	1.0	1.0	1.0	1.0	1.0
tr:I4AY87:I4AY87_HUMAN	1.0	1.0	1.0	1.0	1.0	1.0
sp:P30904:MIF_RAT	0.9	0.98	0.9	0.98	1.0	1.0
sp:P34884:MIF_MOUSE	0.9	0.97	0.89	0.97	1.0	1.0
tr:Q545F0:Q545F0_MOUSE	0.9	0.97	0.89	0.97	1.0	1.0
tr:Q9U228:Q9U228_CAEEL	0.33	0.78	0.33	0.78	0.57	0.73

Nitric oxide synthase, endothelial

Best gene implication in ageing for this target family came from gene F1LQC7 via mapping the annotation from RGD 3186 annotated in RGD 2014-03-11. Annotation GO 7568 (aging) was Inferred from Expression Pattern

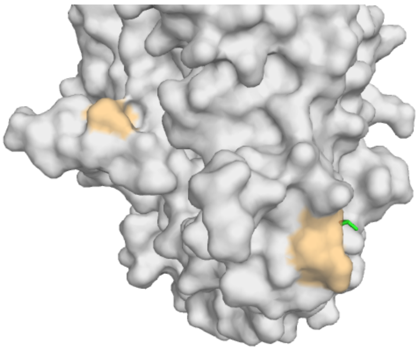

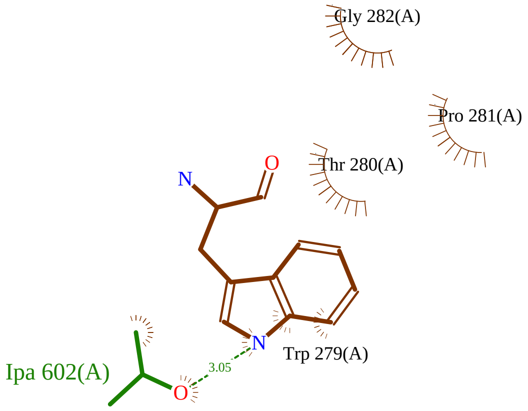

protein	amino acids contacts (binding site)
PDB:1m9m:chainA:P29474	G S W T P G
PDB:1m9q:chainA:P29474	G S W T P G
sp:P29474:NOS3_HUMAN	G S W T P G
tr:F1LQC7:F1LQC7_RAT	G S W T P G
tr:E9Q9X4:E9Q9X4_MOUSE	G S W T P G
sp:P70313:NOS3_MOUSE	G S W T P G
sp:Q27571:NOS_DROME	S S W K S K
sp:P16603:NCPR_YEAST	- - - - - -

	whole protein		domain-based		contact-based	
protein	ident	simil	ident	simil	ident	simil
PDB:1m9m:chainA:P29474	1.0	1.0	1.0	1.0	1.0	1.0
PDB:1m9q:chainA:P29474	1.0	1.0	1.0	1.0	1.0	1.0
sp:P29474:NOS3_HUMAN	1.0	1.0	1.0	1.0	1.0	1.0
tr:F1LQC7:F1LQC7_RAT	0.94	0.98	0.94	0.98	1.0	1.0
tr:E9Q9X4:E9Q9X4_MOUSE	0.86	0.9	0.94	0.99	1.0	1.0
sp:P70313:NOS3_MOUSE	0.94	0.98	0.94	0.99	1.0	1.0
sp:Q27571:NOS_DROME	0.4	0.69	0.49	0.82	0.33	0.57
sp:P16603:NCPR_YEAST	0.14	0.37	0.03	0.07	0.0	0.0

Nos (FBgn0011676) associated phenotypes

chemical resistant, developmental rate defective, dominant, eclosion defective, increased cell size, large body, neuroanatomy defective, partially lethal - majority die, small body, some die during pupal stage

(Information from FlyBase)

Nos (UniProt:Q27571) annotation

Function: Produces nitric oxide (NO) which is a messenger molecule with diverse functions throughout the body. Truncated isoforms (isoform 3-isoform 6) are able to form intracellular complexes with the full length protein and serve as dominant negative inhibitors of the enzyme activity. (PubMed:11526108, PubMed:12804606, PubMed:7568075).
Cofactor: heme
Cofactor: FADNote=Binds 1 FAD. ;
Cofactor: FMNNote=Binds 1 FMN. ;
Enzyme regulation: Stimulated by calcium/calmodulin. (PubMed:7568075).
Developmental stage: Isoform 3 is expressed in larvae only. Isoform 4, isoform 5, isoform 6 and isoform 10 are expressed throughout development from embryos to adults. (PubMed:11526108).

(Information from UniProt)

Oxysterols receptor LXR-beta

Best gene implication in ageing for this target family came from gene P34021 annotated in UniProt release 2014_02. Annotation GO 8340 (determination of adult lifespan) was Inferred from Mutant Phenotype

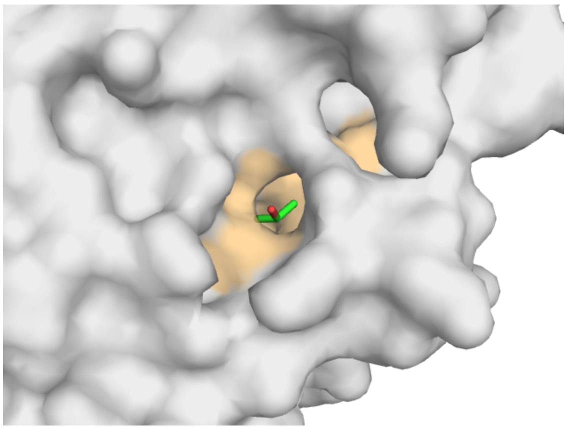

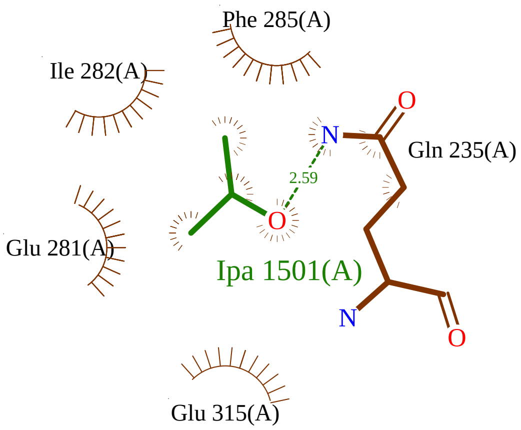

protein	amino acids contacts (binding site)
PDB:1pq6:chainA:P55055	Q E I F E
tr:M0R0K3:M0R0K3_HUMAN	Q E I F E
tr:F1D8N1:F1D8N1_HUMAN	Q E I F E
sp:Q13133:NR1H3_HUMAN	Q E I F E
tr:E9PLL4:E9PLL4_HUMAN	Q E I F E
tr:F1D8P7:F1D8P7_HUMAN	Q E I F E
sp:P55055:NR1H2_HUMAN	Q E I F E
tr:A9UMV6:A9UMV6_RAT	Q E I F E
sp:Q62755:NR1H2_RAT	Q E I F E
tr:Q5I035:Q5I035_RAT	Q E I F E
tr:Q8BP65:Q8BP65_MOUSE	Q E I F E
sp:Q9Z0Y9:NR1H3_MOUSE	Q E I F E
sp:Q60644:NR1H2_MOUSE	Q E I F E
tr:Q91X41:Q91X41_MOUSE	Q E I F E
tr:A4UZ51:A4UZ51_DROME	Q L I F R
sp:P34021:ECR_DROME	Q L I F R
tr:E1JGY2:E1JGY2_DROME	Q L I F R

protein	whole protein ident	simil	domain-based ident	simil	contact-based ident	simil
PDB:1pq6:chainA:P55055	1.0	1.0	1.0	1.0	1.0	1.0
tr:M0R0K3:M0R0K3_HUMAN	0.93	0.93	0.88	0.88	1.0	1.0
tr:F1D8N1:F1D8N1_HUMAN	0.57	0.76	0.76	0.93	1.0	1.0
sp:Q13133:NR1H3_HUMAN	0.57	0.76	0.76	0.93	1.0	1.0
tr:E9PLL4:E9PLL4_HUMAN	0.52	0.69	0.6	0.74	1.0	1.0
tr:F1D8P7:F1D8P7_HUMAN	1.0	1.0	1.0	1.0	1.0	1.0
sp:P55055:NR1H2_HUMAN	1.0	1.0	1.0	1.0	1.0	1.0
tr:A9UMV6:A9UMV6_RAT	0.76	0.82	0.79	0.79	1.0	1.0
sp:Q62755:NR1H2_RAT	0.88	0.94	0.99	1.0	1.0	1.0
tr:Q5I035:Q5I035_RAT	0.57	0.76	0.76	0.93	1.0	1.0
tr:Q8BP65:Q8BP65_MOUSE	0.87	0.93	0.99	1.0	1.0	1.0
sp:Q9Z0Y9:NR1H3_MOUSE	0.58	0.76	0.77	0.93	1.0	1.0
sp:Q60644:NR1H2_MOUSE	0.87	0.93	0.99	1.0	1.0	1.0
tr:Q91X41:Q91X41_MOUSE	0.58	0.76	0.77	0.93	1.0	1.0
tr:A4UZ51:A4UZ51_DROME	0.17	0.36	0.31	0.62	0.6	0.55
sp:P34021:ECR_DROME	0.17	0.34	0.31	0.62	0.6	0.55
tr:E1JGY2:E1JGY2_DROME	0.17	0.34	0.31	0.62	0.6	0.55

EcR (FBgn0000546) associated phenotypes

RU486 conditional, cell death defective, cell shape defective, circadian rhythm defective, conditional, courtship behavior defective, decreased cell death, decreased cell size, developmental rate defective, dominant, feeding behavior defective, heat sensitive, lethal - all die before end of P-stage, lethal - all die before end of first instar larval stage, lethal - all die before end of prepupal stage, lethal - all die before end of pupal stage, locomotor behavior defective, long lived, male fertile, memory defective, mitotic cell cycle defective, neuroanatomy defective, partially, partially lethal - majority die, short lived, size defective, sleep defective, small body, somatic clone, some die during first instar larval stage, some die during pharate adult stage, some die during prepupal stage, some die during pupal stage, some die during third instar larval stage

(Information from FlyBase)

EcR (UniProt:P34021) annotation

Function: Receptor for ecdysone. Binds to ecdysone response elements (ECRES) following ecdysone-binding, and recruitment of a complex containing the histone methyltransferase trr, leads to activate transcription of target genes. (PubMed:1913820).
Subunit: Heterodimer of USP and ECR. Only the heterodimer is capable of high-affinity binding to ecdysone. Interacts with trr in an ecdysone-dependent manner. (PubMed:14603321, PubMed:8247157).
Subcellular location: Nucleus (PROSITE- ProRule:PRU00407, PubMed:1913820).
Tissue specificity: Isoform B1 predominates over isoform A in larval tissues, imaginal histoblast nests and midgut islands. Isoform A predominates over B1 in imaginal disks, and the larval prothoracic gland. (PubMed:1913820).
Developmental stage: In the salivary glands of mid instar larvae levels increase during puff stage 1 at 86-94 hours of development then remain relatively constant until the premetamorphic pulse of ecdysone in late larvae. Levels diminish dramatically from puff stage 7 onwards. Levels increase in the prepupal period during puff stage 13-14, the level remains stable until stage 21. A decrease in levels at puff stage 7 is also seen in the Malpighian tubules and less dramatically in the fat body and gut. In the wing disk the relatively low level remains unchanged. (PubMed:1913820, PubMed:8223281).

(Information from UniProt)

Focal adhesion kinase 1

Best gene implication in ageing for this target family came from gene Q05397 via mapping the annotation from Ensembl ENSG00000169398 via mapping the annotation from EntrezGene 5747 via mapping the annotation from GenAgeHuman 0166 annotated in GenAge release 17.

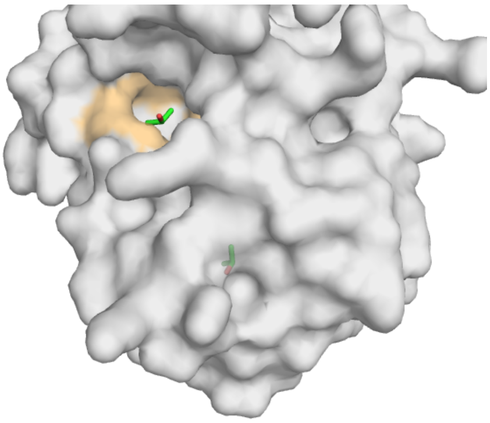

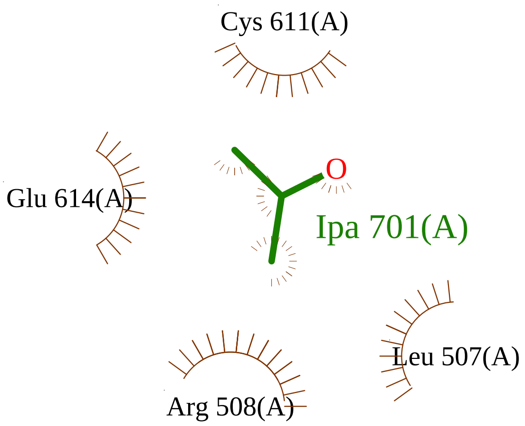

protein	amino acids contacts (binding site)									
PDB:4ebv:chainA:Q05397	L	R	W	V	C	E	F	I	R	L	W
PDB:4i4f:chainA:Q05397	L	R	W	V	C	E	F	I	R	L	W
tr:E9PEI4:E9PEI4_HUMAN	L	R	W	V	C	E	F	I	R	L	W
tr:H0YB16:H0YB16_HUMAN	L	R	W	V	C	E	F	I	R	L	W
tr:Q8N9D7:Q8N9D7_HUMAN	L	R	W	V	C	E	F	I	R	L	W
tr:B4DH13:B4DH13_HUMAN	L	R	W	V	C	E	F	I	R	L	W
tr:H0YBZ1:H0YBZ1_HUMAN	L	R	W	V	C	E	F	I	R	L	W
tr:Q8IYN9:Q8IYN9_HUMAN	L	R	W	V	C	E	F	I	R	L	W
sp:Q05397:FAK1_HUMAN	L	R	W	V	C	E	F	I	R	L	W
tr:J3QT16:J3QT16_HUMAN	L	R	W	V	C	E	F	I	R	L	W
tr:E7ESA6:E7ESA6_HUMAN	L	R	W	V	C	E	F	I	R	L	W
tr:R9PXT6:R9PXT6_RAT	L	R	W	V	C	E	F	I	R	L	W
sp:P34152:FAK1_MOUSE	L	R	W	V	C	E	F	I	R	L	W
tr:Q2TGI0:Q2TGI0_DROME	L	R	W	V	C	E	F	L	R	L	W
tr:Q9U5Y2:Q9U5Y2_DROME	L	R	W	V	C	E	F	L	R	L	W
tr:E1JGM8:E1JGM8_DROME	L	R	W	V	C	E	F	L	R	L	W
tr:B8A418:B8A418_DROME	L	R	W	V	C	E	F	L	R	L	W
tr:Q5BIG9:Q5BIG9_DROME	L	R	W	V	C	E	F	L	R	L	W
tr:Q0E917:Q0E917_DROME	L	R	W	V	C	E	F	L	R	L	W
tr:Q9U531:Q9U531_DROME	L	R	W	V	C	E	F	L	R	L	W
tr:Q9U472:Q9U472_DROME	L	R	W	V	C	E	F	L	R	L	W
tr:B7YZL9:B7YZL9_DROME	L	R	W	V	C	E	F	L	R	L	W
tr:Q8T879:Q8T879_CAEEL	L	R	W	V	C	E	W	I	R	P	W
tr:Q95YD4:Q95YD4_CAEEL	L	R	W	V	C	E	W	I	R	P	W

protein	whole protein ident	simil	domain-based ident	simil	contact-based ident	simil
PDB:4ebv:chainA:Q05397	1.0	1.0	1.0	1.0	1.0	1.0
PDB:4i4f:chainA:Q05397	1.0	1.0	1.0	1.0	1.0	1.0
tr:E9PEI4:E9PEI4_HUMAN	0.63	0.64	1.0	1.0	1.0	1.0
tr:H0YB16:H0YB16_HUMAN	0.68	0.68	1.0	1.0	1.0	1.0
tr:Q8N9D7:Q8N9D7_HUMAN	0.67	0.67	1.0	1.0	1.0	1.0
tr:B4DH13:B4DH13_HUMAN	0.65	0.65	1.0	1.0	1.0	1.0
tr:H0YBZ1:H0YBZ1_HUMAN	0.66	0.66	1.0	1.0	1.0	1.0
tr:Q8IYN9:Q8IYN9_HUMAN	0.96	0.96	1.0	1.0	1.0	1.0
sp:Q05397:FAK1_HUMAN	1.0	1.0	1.0	1.0	1.0	1.0
tr:J3QT16:J3QT16_HUMAN	0.99	0.99	1.0	1.0	1.0	1.0
tr:E7ESA6:E7ESA6_HUMAN	0.96	0.96	1.0	1.0	1.0	1.0
tr:R9PXT6:R9PXT6_RAT	0.97	0.99	1.0	1.0	1.0	1.0
sp:P34152:FAK1_MOUSE	0.94	0.96	1.0	1.0	1.0	1.0
tr:Q2TGI0:Q2TGI0_DROME	0.16	0.23	0.67	0.89	0.91	1.0
tr:Q9U5Y2:Q9U5Y2_DROME	0.3	0.62	0.67	0.89	0.91	1.0
tr:E1JGM8:E1JGM8_DROME	0.3	0.62	0.67	0.89	0.91	1.0
tr:B8A418:B8A418_DROME	0.3	0.62	0.67	0.89	0.91	1.0
tr:Q5BIG9:Q5BIG9_DROME	0.3	0.62	0.66	0.88	0.91	1.0
tr:Q0E917:Q0E917_DROME	0.3	0.62	0.67	0.89	0.91	1.0
tr:Q9U531:Q9U531_DROME	0.3	0.62	0.67	0.89	0.91	1.0
tr:Q9U472:Q9U472_DROME	0.3	0.62	0.67	0.89	0.91	1.0
tr:B7YZL9:B7YZL9_DROME	0.25	0.52	0.67	0.89	0.91	1.0
tr:Q8T879:Q8T879_CAEEL	0.21	0.53	0.54	0.84	0.82	0.87
tr:Q95YD4:Q95YD4_CAEEL	0.21	0.55	0.54	0.84	0.82	0.87

Fak (FBgn0020440) associated phenotypes

neuroanatomy defective, neurophysiology defective

(Information from FlyBase)

Heterogeneous nuclear ribonucleoprotein L

Best gene implication in ageing for this target family came from gene Q0E914 annotated in UniProt release 2014_02. Annotation GO 8340 (determination of adult lifespan) was Inferred from Mutant Phenotype

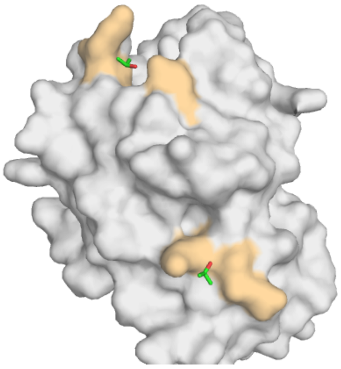

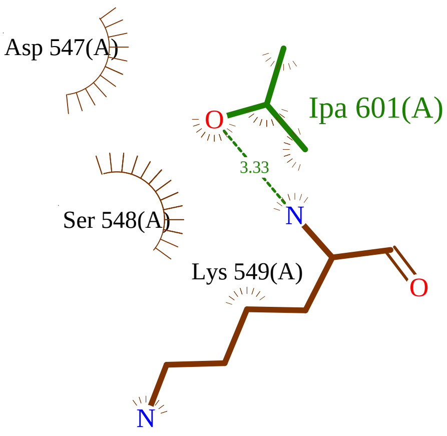

protein	amino acids contacts (binding site)
PDB:3s01:chainA:Q8R081	M Y S K D S K
tr:Q6NTA2:Q6NTA2_HUMAN	M Y S K E S K
sp:P14866:HNRPL_HUMAN	M Y S K E S K
tr:Q5U1Y5:Q5U1Y5_RAT	M Y S K D S K
tr:F2Z3R2:F2Z3R2_RAT	M Y S K D S K
tr:F1LQ48:F1LQ48_RAT	M Y S K D S K
tr:G3UY38:G3UY38_MOUSE	M Y S K D S K
sp:Q8R081:HNRPL_MOUSE	M Y S K D S K
tr:Q0E914:Q0E914_DROME	M Y S K S N I
tr:A1ZBN3:A1ZBN3_DROME	M Y S K S N I
tr:Q8IHH3:Q8IHH3_DROME	M Y S K S N I
tr:D5AEJ9:D5AEJ9_DROME	M Y S K S N I
tr:Q7JMZ7:Q7JMZ7_DROME	M Y S K S N I
tr:Q6NND8:Q6NND8_DROME	M Y S K S N I
tr:Q95QR5:Q95QR5_CAEEL	M Y S H E T V

	whole protein		domain-based		contact-based	
protein	ident	simil	ident	simil	ident	simil
PDB:3s01:chainA:Q8R081	1.0	1.0	0.99	1.0	1.0	1.0
tr:Q6NTA2:Q6NTA2_HUMAN	0.95	0.96	0.98	1.0	0.86	0.93
sp:P14866:HNRPL_HUMAN	0.98	0.99	0.98	1.0	0.86	0.93
tr:Q5U1Y5:Q5U1Y5_RAT	0.42	0.42	1.0	1.0	1.0	1.0
tr:F2Z3R2:F2Z3R2_RAT	1.0	1.0	1.0	1.0	1.0	1.0
tr:F1LQ48:F1LQ48_RAT	0.94	0.94	1.0	1.0	1.0	1.0
tr:G3UY38:G3UY38_MOUSE	0.78	0.78	1.0	1.0	1.0	1.0
sp:Q8R081:HNRPL_MOUSE	1.0	1.0	1.0	1.0	1.0	1.0
tr:Q0E914:Q0E914_DROME	0.15	0.29	0.4	0.76	0.57	0.67
tr:A1ZBN3:A1ZBN3_DROME	0.16	0.38	0.4	0.76	0.57	0.67
tr:Q8IHH3:Q8IHH3_DROME	0.16	0.39	0.4	0.76	0.57	0.67
tr:D5AEJ9:D5AEJ9_DROME	0.17	0.43	0.4	0.76	0.57	0.67
tr:Q7JMZ7:Q7JMZ7_DROME	0.25	0.49	0.4	0.76	0.57	0.67
tr:Q6NND8:Q6NND8_DROME	0.25	0.5	0.4	0.76	0.57	0.67
tr:Q95QR5:Q95QR5_CAEEL	0.25	0.5	0.28	0.72	0.43	0.69

sm (FBgn0003435) associated phenotypes

feeding behavior defective, long lived, neuroanatomy defective, partially lethal - majority die, short lived, some die during pupal stage

(Information from FlyBase)
